# Nurse Educators' Conceptions of How They Facilitate Critical Thinking in Bachelor Nursing Students: A Phenomenographic Study

**DOI:** 10.1111/jan.70327

**Published:** 2025-10-28

**Authors:** Frida Westerdahl, Anne Wennick, Gunilla Borglin, Elisabeth Carlson

**Affiliations:** ^1^ Department of Care Science, Faculty of Health and Society Malmö University Malmö Sweden; ^2^ Department of Bachelor's in Nursing Lovisenberg Diaconal University College Oslo Norway

**Keywords:** conceptions, critical thinking, nurse educators, nursing, nursing education, nursing students, phenomenography, qualitative research

## Abstract

**Aim:**

To describe the variation in nurse educators' conceptions of how they facilitate critical thinking in bachelor nursing students.

**Design:**

Qualitative study with a phenomenographic approach.

**Methods:**

Data was collected through twenty‐six semi‐structured interviews with nurse educators conducted in Sweden between March and June 2024.

**Results:**

The result of this study can be understood as five descriptive categories: Creating a safe and trustful relationship with the students, Encouraging a dialogue with the students, Using space as a tool, Using artefacts as a tool, and Using oneself as a tool.

**Conclusion:**

The conclusion is that the facilitation of critical thinking needs to be based on a safe and trustful relationship between educators and students. Without this relationship, it is not possible to establish the central dialogue, where the educator can facilitate critical thinking through asking counterquestions and provoking the students.

**Implication for the Profession:**

To become critical thinkers, the students need to put their knowledge and assumptions in a new light and question them. Here, the educator has a vital role in being the guide and facilitator.

**Impact:**

The result indicated that it is vital for the educators to build a safe relationship with the students. The relationship is a precondition for the facilitative dialogue where the educators can ask reflective and provoking questions to stimulate critical thinking. Future nurses need to be prepared with critical thinking to enable evidence‐based clinical decisions both during clinical practice as well as when being registered nurses.

**Reporting Method:**

SRQR guidelines.

**Patient and Public Involvement:**

This study did not include patient or public involvement in its design, conduct, or reporting.

## 1 Introduction

Critical thinking is viewed as one of the most vital goals of higher education, regardless of subject or discipline (Golden 2023). To implement critical thinking within disciplines is a major educational challenge in the 21st century, and it is, furthermore, seen as a necessary skill for employability (Sellars et al. 2018). Critical thinking is explained as a person's ability to make well‐considered as well as defendable judgments and decisions, that is, knowing what to believe in and do in a certain situation (Ennis 1991). In nursing, critical thinking is identified as an essential factor for high‐quality care and provides an opportunity for nurses to take evidence‐based clinical decisions followed by an evaluation of the outcome for the patients (Falcó‐Pegueroles et al. 2021). Therefore, critical thinking needs to be promoted and developed throughout the undergraduate nursing programme by competent nurse educators (hereafter educators) to prepare future nurses for their upcoming professional responsibilities (Noone and Seery 2018).

## 2 Background

In today's demanding clinical environment, nurses need to be well equipped with critical thinking skills and able to apply them to deliver safe and optimal care for each patient. Possessing critical thinking also enables nurses to work autonomously and independently in health care, which could develop the profession (Chan 2019). Here, educators play an important role in developing, planning, and assessing course content with the aim of facilitating critical thinking in future registered nurses. It is a skill that requires guidance and support to be developed and should be implemented through active learning strategies (Nelson 2017). As stated by Chan (2013), the facilitation of critical thinking needs to be introduced early in the nursing education to achieve the best impact on the students' clinical enactment. Further, the curricula should include a variety of teaching strategies to give the students several opportunities throughout their education to learn and practice critical thinking, which enables students to move from simple to more complex ways of thinking necessary in delivering care (Von Colln‐Appling and Giuliano 2017). From the perspective of nursing students, they highlight the importance of small size groups and the possibility to interact with their educators face to face in the facilitation of critical thinking. The students further describe critical thinking as difficult to be taught since it is primarily based on experiences; however, it could be aided by simulations and the possibility to practice it in the clinical context (Wong and Kowitlawakul 2020). Connected to this, it has also been shown that individual factors such as empathy and emotional intelligence are closely connected to the development of critical thinking in nursing students (Zarzycka and Gesek 2022).

Critical thinking is said to be a dynamic and complex ability constituted by both strategic and attitudinal skills aiming at a particular purpose (Falcó‐Pegueroles et al. 2021). Facione (1990) argues that critical thinking is the analysis and evaluation of a situation resulting in a well‐established judgement. It can also be described as a consecutive process consisting of four steps: collecting information, investigation, evaluation, and lastly, problem solving (Chan 2013). The term critical thinking can be seen in contrast to the adjacent term reflective thinking, which refers to the process of assessing and reviewing past experiences, knowledge, and events when new decisions are made. In this sense, reflective thinking has an impact on the ability to think critically but solely from a retrospective point of view (Akpur 2020; Ghanizadeh 2017). From the nursing student's point of view, critical thinking is outlined as a skill that requires them to think both logically and for themselves, questioning perceptions of others as well as discussing the truthfulness of evidence and sources (Chan 2019). Further, critical thinking enables the students to make the link between theory and practice, which in turn is seen as a foundation for conducting sound and rational clinical decisions (Wong and Kowitlawakul 2020). However, there are several definitions of critical thinking, and none of them is superior to the others since critical thinking is contextually bound (Alfaro‐LeFevre 2019). In this study, we take the definition suggested by Scheffer and Rubenfeld's (2000) Delphi study as our point of departure, as it has a nursing origin and focuses on the clinical aspects of critical thinking:

Critical thinking in nursing is an essential component of professional accountability and quality nursing care. Critical thinkers in nursing exhibit these habits of the mind: confidence, contextual perspective, creativity, flexibility, inquisitiveness, intellectual integrity, intuition, open‐mindedness, perseverance, and reflection. Critical thinkers in nursing practice the cognitive skills of analyzing, applying standards, discriminating, information seeking, logical reasoning, predicting and transforming knowledge. (Scheffer and Rubenfeld 2000, 357)

The rationale for this study is based on a preceding scoping review by Westerdahl (2022) as well as studies by Nelson (2017), Von Colln‐Appling and Giuliano (2017) and Chan (2013), stating that previous studies focused on which teaching strategies are implemented in nursing educations to facilitate critical thinking, for example, problem‐based learning (PBL), cases and simulations. However, studies seemed to be lacking concerning how the educators facilitate critical thinking during the teaching strategies in a combination with that only three out of 19 studies included educators as population. Further, research needs to discover productive strategies to develop critical thinking and not only measure the ability to think critically before and after an educational intervention (Falcó‐Pegueroles et al. 2021). Since critical thinking is an important skill for future nurses to be able to pursue nursing based on evidence as well as making sound clinical decisions more attention needs to be drawn to how educators facilitate critical thinking in bachelor nursing students.

## 3 Aim

The aim was to describe the variation in nurse educators' conceptions of how they facilitate critical thinking in bachelor nursing students.

## 4 Method

This qualitative study employed a phenomenographic approach. Phenomenography is particularly useful when exploring questions related to education and learning (Marton 1986), and thus is in line with the aim of this study. The approach focuses on the qualitatively different ways a phenomenon in the surrounding world can be described, analysed, and understood. A distinction is made between the first‐ and second‐order perspectives. Where the first‐order perspective focuses on the world as such and how it is described, the second‐order perspective makes statements about people's ideas and experiences of the world (Marton 1981). It is the latter perspective that is in focus in a phenomenographic study.

Since phenomenography has its roots in education, it is deemed appropriate to use in research on nursing education, as well as on approaches to teaching and different teacher roles (Stenfors‐Hayes et al. 2013; Whitfield et al. 2023). It is further applicable in educational research since the focus is on exploring the various ways a phenomenon can be described and experienced (Sjöström and Dahlgren 2002; Stenfors‐Hayes et al. 2013; Whitfield et al. 2023). As explored in this study as nurse educators' varying ways of facilitating critical thinking.

This manuscript was written according to the Standards for Reporting Qualitative Research (SRQR), as described by O'Brien et al. (2014).

### 4.1 Study Setting

In Sweden, the undergraduate nursing education is three years and results in a bachelor's degree in nursing (180 European Credit Transfer System [ECTS]). The educators are, in most cases, registered nurses with at least a one‐year master's in nursing science and 15 ECTS in Teaching and Learning in Higher Education. Other professions with adjacent academic subjects can also teach at the nursing programme, for example, physiotherapists and physicians.

### 4.2 Recruitment and Participants

A purposeful sampling method was used to reach a varied population, as recommended by Han and Ellis (2019). The inclusion criterion was that the participants should hold a teaching position at a nursing programme. As a first step in the recruitment process, all twenty‐four heads of departments at nursing programmes in Sweden were contacted for approval to approach educators at their universities, of which twenty gave their permission to pursue with recruitment. This was followed by gatekeepers, who were either persons known by the research team or administrators found through the universities' websites, sending out information about the study through e‐mail. Those who were interested in participation contacted the first author to decide on a day for an interview at the educators' convenience. Twenty‐six educators, representing fourteen universities in Sweden, gave their informed consent to participate in the study (Table 1).

**TABLE 1:** An overview of the participants' demographics.

Participants (*n*)	26
Professional title (*n*)	
Registered nurse	24
Nutritionist	1
Physician	1
Academic title (*n*)	
Professor	1
Associate professor	1
Senior lecturer	6
Lecturer	14
PhD student	4
Educator at a nursing programme	
In years (mean)	10

### 4.3 Data Collection

**FIGURE 1:**
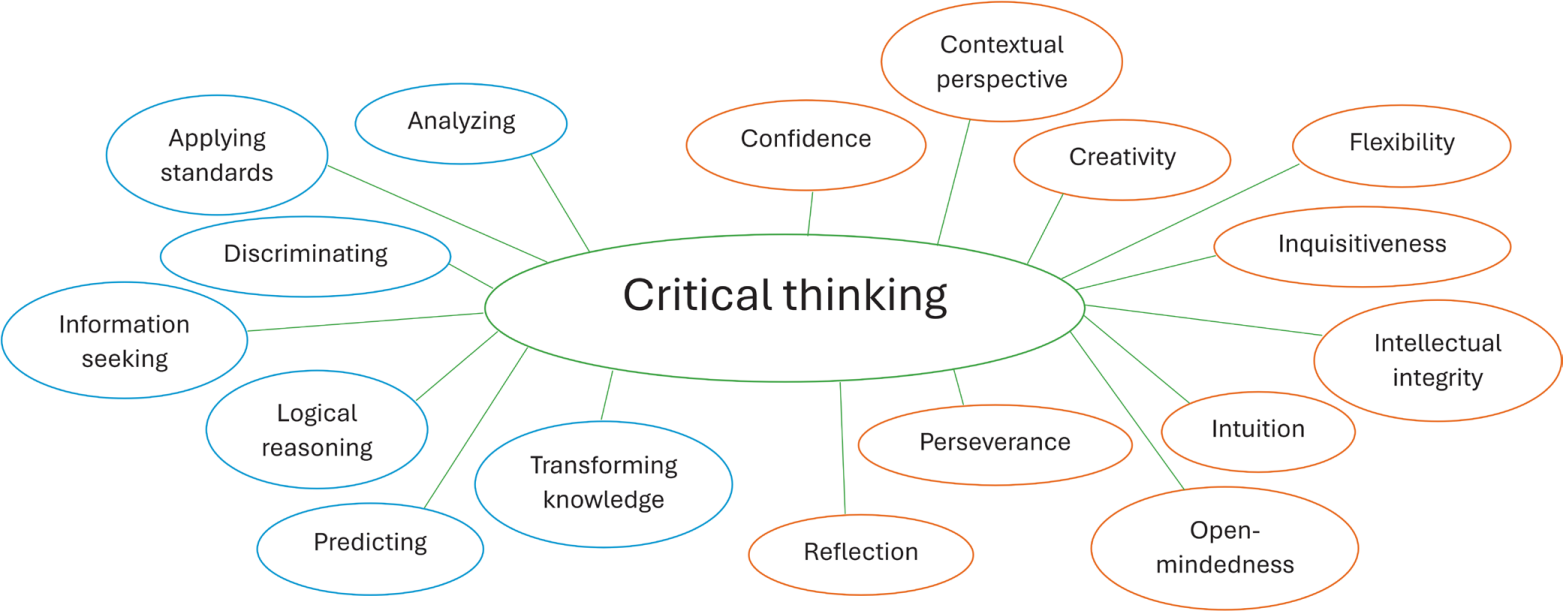
Mind map based on Scheffer and Rubenfeld's (2000) definition of critical thinking.

Data was collected through individual semi‐structured interviews using Zoom Workplace (Version 6.1.11). All interviews were conducted between March and June 2024 in Sweden by the first author (FW), who is well versed in qualitative methods. To test the interview guide, two pilot interviews were realised, resulting in no necessary adjustments being needed. The pilot interviews were held with colleagues of the authors who met the inclusion criteria, but to avoid bias, these interviews were not included in the data analysis. As described in the background, the current study is based on Scheffer and Rubenfeld's (2000) definition of critical thinking in nursing. The definition was translated into Swedish by the authors, included in the information letter, and converted to a mind map (see Figure 1). The interviews started with the first author having an everyday conversation with the educators to get them comfortable with the situation and inviting possible questions. After this, the first author's screen was shared, making the mind map visible, followed by the opening question ‘What are your thoughts after reading Scheffer and Rubenfeld's definition of critical thinking in nursing?’. The mind map was observable throughout the conversations. The interviews continued with a dialogue based on the educators' responses, as suggested by Sjöström and Dahlgren (2002) and Han and Ellis (2019), and probing questions like ‘Can you give an example’ and ‘Can you explain that further’. The conversations were rounded off with the question ‘Is there anything concerning how you facilitate critical thinking in nursing students that you would like to add?’. All interviews were recorded in Zoom Workplace (Version 6.1.11) and transcribed through speech recognition in Microsoft Word (version 2501). A Dictaphone was used for backup. The interviews lasted between 33 and 66 min (mean 47 min). After twenty‐six interviews, no new data from the interviews emerged; therefore, the group of authors decided to end the data collection. This number goes approximately in line with the recommended 15–20 interviews indicated by Balding et al. (2024) who further claim that an exact amount of participants is hard to outline since it is dependent on the saturation of data.

Twenty out of the twenty‐six transcripts were listened to and corrected for errors by the first author, as recommended by Eftekhari (2024). As for the remaining six transcripts, the original transcripts were not transcribed verbatim but analyzed by listening repeatedly to the voice recordings, as suggested by Halcomb and Davidson (2006). This was pursued with the aim of verifying the inductive findings in the first twenty interviews and transcripts. As explained by Hyde (2000) a portion of the data is archived, and these are later tested against the tentative findings as a confirmation. All twenty‐six transcripts were included in the analysis. Since member checking is not the common way of ensuring trustworthiness in phenomenography (Balding et al. 2024), the transcripts were not returned to the participating educators.

### 4.4 Data Analysis

The aim of a phenomenographic data analysis is to present the qualitatively different ways a phenomenon can be described, that is, the variation of a phenomenon (Han and Ellis 2019). The data in this study was analyzed inductively according to the seven steps outlined by Dahlgren and Fallsberg (1991). In the first step, *familiarisation*, all four authors read two transcripts each to get a first impression of the data, which was discussed in the group. In the next step, *condensation*, the first author read all twenty‐six transcripts and marked text excerpts corresponding to the aim. This was followed by *comparison* and *grouping*, where marked text excerpts were compared and arranged according to similarities and differences. The fifth step, *articulating*, was realised through formulating text describing the findings, followed by step six, *labelling*, where each descriptive category, which is the meanings and thoughts that is, the conceptions represented on a collective level (Balding et al. 2024; Stenfors‐Hayes et al. 2013), was named according to its content. Lastly, during *contrasting*, the descriptive categories were compared to clarify differences and similarities. The first and last author had the major responsibility for steps three through seven. However, the entire research team had regular meetings along the process, for GB and AW to contribute with insights and new perspectives, for example, if the categories were distinct enough or should be collapsed. Further, as recommended by Stenfors‐Hayes et al. (2013) an iterative process was adopted through reading the transcripts and going through the steps of the analysis process repeatedly. When this had been applied, a consensus was reached in the group of authors that no more conceptions existed among the transcripts and that the descriptive categories were not overlapping. An example of the analysis process and the included steps is visualised in Table 2.

**TABLE 2:** Examples from the data analysis process.

Familiarisation (visualised through citation from transcript)	Condensation	Comparison	Grouping	Articulating	Labelling	Contrasting
*What should I say, not to be superior, not to say like don't you understand that, that is not reasonable and so on, but my expectations is that themselves when we sit and discuss will twist and turn things and reason*	Through being humble, not humiliate when they don't know the answer	Open and safe environment created by the educator	Environment/relation	The educators realised this through positive feedback, encouragement and emphasising that everyone's contribution was equally important	Creating a safe and trustful relationship with the students	A distinction was made that this descriptive category focused on the relationship between educator and students and was seen as a precondition for the dialogue
*Well i ask, ask maybe stupid questions maybe one can say why or if they say it is like this… is it really so?… how do we know that or why is it like this or what can it mean so that they have to reason around if it is true what I say or if it is just my conceptions so they have to think twice*	Through reflective questions, motivate their thoughts	Dialogue/questioning facilitated by the educator	Dialogue between educator and students	The educator encouraged a dialogue through reflective questioning or asking the students if something could be executed differently or what happens if nothing is done	Encouraging a dialogue with the students	A distinction was made that this descriptive category focused on the dialogue where a relationship was necessary to establish first
*Deliberately leave openings in the cases or that it is unclear cases, that they are unclearly written in some instances so it can be interpreted in different ways and that is what we are aiming for, that the students interpret it differently*	Through unclear cases which create reflection	Depart in cases and real situations	Material/artefacts	Patient cases could be either fictive or based on authentic clinical situations, but were purposely unclear without exact answers	Using artefacts as a tool	A distinction was made between the different tools used, to clarify what strategies belonged to space, artefacts and the educator oneself

### 4.5 Ethical Considerations

All participating educators received before the interviews an information letter including information about the study, ethical considerations, and the researchers' academic positions as well as affiliated universities. Signed informed consent was collected before the interviews, and the educators had the right to withdraw at any stage of the research process without specifying why. The educators had further the possibility to ask questions regarding the study both before signing informed consent as well as before the interviews took place. To minimise the risk for power imbalances, only one researcher was present during the interviews, and the educators themselves decided on a day as well as where they wanted to be placed during the interviews. The e‐meeting link was also encrypted to avoid interference from unauthorised third‐party participants. Data was handled confidentially and kept in a secure computer storage only accessible by the first author. These ethical considerations are in line with the Helsinki Declaration (World Medical Association 2024). The study did not gather any sensitive personal information or data and thereby did not fall under the Swedish Ethics Review Act (2003:460).

### 4.6 Methodological Considerations

To ensure consistency in data collection, all interviews were executed by the same researcher and preceded by pilot interviews (Whitfield et al. 2023). However, the analysis involved all four authors, that is, investor triangulation, as well as two researchers had the main responsibility for the analysis and repeatedly discussed with the other researchers throughout the process. This was pursued with the aim of reaching credibility and is highlighted by Stenfors‐Hayes et al. (2013) and Whitfield et al. (2023). Credibility was further enhanced through a detailed and transparent description of the methods used for data collection and analysis in combination with the presentation of verbatim quotations in the findings section, as recommended by Balding et al. (2024) and Stenfors‐Hayes et al. (2013). In terms of transferability, the context was presented in a thorough way; however, as pointed out by Balding et al. (2024) and Sin (2010) phenomenographic studies are unique and context‐specific, claiming that transferability is neither desired nor possible. Lastly, to address confirmability and reflexivity, interpretative awareness was adopted through the researchers being transparent about their own understandings and experiences of the phenomenon studied (Balding et al. 2024; Whitfield et al. 2023). All four authors of the study are active as educators in different nursing programmes and thereby have experience in the research area. Therefore, during the interviews, the first author tried to be influenced by her own educator role as little as possible and avoid personal responses, as well as all authors adopting an open mind during the analysis process to not be affected by pre‐assumptions. Further, the first author pursued the interviews in the role of PhD student, despite having experience in education, so as not to jeopardise the richness of descriptions in the interviews.

Another methodological consideration was the fact that the data collection was pursued through online video interviewing. As pointed out by Saarijärvi and Bratt (2021), video interviews have the advantage of possibly increasing the study population since distance is not a problem and travel issues are avoided. Furthermore, it is still possible for the interviewer to see the participants and their body language as well as facial expressions during data collection (ibid.). None of the participants in the current study had any objections to video interviewing, probably because educators are used to digital meetings and teaching in their everyday work.

## 5 Findings

Nurse educators' conceptions of how they facilitate critical thinking in bachelor nursing students can be understood through five descriptive categories: *Creating a safe and trustful relationship with the students*, *Encouraging a dialogue with the students*, *Using space as a tool*, *Using artefacts as a tool*, and *Using oneself as a tool*.

To enhance the confirmability of the findings, the descriptive categories are illustrated below with quotations from the original transcripts, marked ‘Educator’ and a number to show which interview the extract originates from without identifying the individual participant.

### 5.1 Creating a Safe and Trustful Relationship With the Students

The educators described how they needed to create a safe and trustful relationship with the students to be able to facilitate critical thinking. They realised this through positive feedback, active listening, encouragement, and a curious interest in the students' viewpoints. This was accomplished by focusing on the students' strengths, boosting them, and making them believe in themselves. However, it was emphasised that their facial expressions and body language had to harmonise with what they said so as not to lose the students' confidence in their educator. When giving feedback on assignments, one strategy used by the educators was to state that it was the assignment that had deficiencies, not the students themselves. Similarly, they made a point of not claiming that an assignment or a statement was poor or wrong, claiming instead that it was interesting and guiding the student regarding how it could be improved.

I try to be curious, cheerful and open. Showing that I'm interested in what they are saying, in their knowledge, and I'd rather try to be someone they can discuss with than an authoritarian person who tells them how things should be done and gives directions. (Educator #13)

The educators also built a safe relationship with the students through getting to know them, learning their names and emphasising that everyone's contribution to the dialogue was equally important. Lastly, building this relationship could also be done through having an everyday and informal conversation with the students before the teaching strategies started, for example, during the assessment of clinical practice—that is, listening to the students' experiences and thoughts on the clinical practice before going into the formal assessment.

The one who describes or shares something should feel that, irrespective of whether it is I or someone else who asks the questions or raises something, they are acknowledged and listened to. (Educator #8)

### 5.2 Encouraging a Dialogue With the Students

The educators described how fundamental it is to encourage a dialogue in order to facilitate critical thinking in nursing students. Some educators initiated and guided the dialogue themselves through asking the students reflective questions. Others used the opposite strategy, that is, they let the students start the dialogue and came in later to promote it through developing questions or discussing with the students. Either way, the educators informed the students beforehand that the teaching strategy included dialogue and that everyone was expected to take part. Some educators also set up rules in collaboration with the students, for example, that confidentiality should be applied. Furthermore, how to structure the dialogue differed among the educators; some had predefined questions or a guide that in some cases was shared among the students beforehand. Others asked questions ad hoc as the dialogue proceeded.

I inform the students, I talk to them already at the first meeting before the course starts, [telling them] that in this course we will reflect a lot, we will ask challenging questions, we want you to get involved in the discussion. I try to prepare them beforehand. (Educator #16)

During the dialogue, the educators asked the students reflective questions: What? Why? How? When? Who? and Where? By doing so the educators wanted the students to arrive at new or changed perspectives and understand how things are connected. They also asked the students if something could be executed in different ways or what would happen if nothing was done, as well as requesting that the students give reasons for their actions and thoughts, making them think of arguments and articulate their reasoning. Moreover, the educators made sure that everyone's opinion was raised even if the answer was wrong, because that in itself could be a catalyst for the dialogue. In the same way, the educators rarely gave an answer to questions raised and instead asked the students about their opinion, explaining to the students why they did not give answers.

I usually say to them [the students]: often when you ask a question you want an answer. But I seldom give a direct answer because I want you to learn it yourself, that you should have an argument for why you do something in a certain way. Not just doing things on routine, but really reflecting on what the best strategy is in this situation. (Educator #13)

Another strategy used was to provoke and make the students react through exaggerating a situation or challenging the students by delivering arguments which turned their way of thinking around. It could also be done through contradicting the students' thoughts and reasoning, or playacting a patient or clinical situation in the dialogue to make the patient's perspective visible for the students.

They [the students] are talking and I can place myself away from them and just chill but when I notice, I find that it's rather funny, that they are starting to talk about things reflecting a lot of prejudices, I allow them to go on for a while and then I interrupt and pose a really tricky question that makes them lose their footing because I'm punctuating their reflections. (Educator #9)

During the dialogues, the educators made sure that all students were included through inviting all of them to speak, and especially the ones who were quiet, but also through clearly stating that everyone's standpoint was welcome and that the educators would help them through their reasoning. Another strategy was making activity in the dialogue and reflective inputs part of the examination. That meant that it was stated in the course curriculum that it was mandatory for the students to be critical thinkers in order to receive a passing grade, for example, in a compulsory seminar.

### 5.3 Using Space as a Tool

One way to facilitate critical thinking, described by the educators, was using the physical or digital space to create a dialogue with the students. Some educators arranged their teaching in a physical classroom since it enabled them to see the students' body language, reactions, and emotions, something that they claimed was necessary for the dialogue. However, other educators used digital meeting tools for teaching because that made students dare to open up, and different chat functions could be used for interaction for those who thought it was hard to speak publicly.

Zoom lectures, for many it can be easier to have that kind of dialogue because you're more invisible online and maybe dare to ask the critical questions, instead of being in a large lecture hall with a lot of people looking at you. (Educator #10)

Regardless of being in a physical or a digital space, the educators avoided large lectures, instead arranging the teaching in smaller groups in order to facilitate critical thinking. The educators claimed that in a smaller group they could see all students, give them attention and address them in the dialogue.

I feel that in the smaller groups I can come closer to the students and this enhances the facilitation of critical thinking in another way, since more students dare to speak and I can actually also approach every student to include them in the dialogue. (Educator #3)

The educators also used the furniture in the physical space, through placing chairs in a circle to allow everyone see each other during the dialogue. Another strategy was to arrange smaller groups with tables, chairs and a white board where the students could write their analysis of the assignment. Meeting in a physical room also enabled the educators to walk around among the students, show their presence and involve themselves in the dialogues through asking questions to facilitate critical thinking.

### 5.4 Using Artefacts as a Tool

The educators described how they applied different artefacts; that is, objects used to assist teaching, in the facilitation of critical thinking, for example, scientific articles, patient cases, and written reflections. When the students and educators reviewed scientific articles, they used validated quality assessment protocols as a guide to make the students take a critical stance towards choices pursued in the research process. Further, the educators distributed articles on similar topics but with varying methodological standpoints or varying quality to exemplify that researchers can arrive at different results, and they reflected together with the students on why this was the case. The students could also be asked to search for scientific articles to solve nursing problems or to find evidence behind nursing actions.

At article seminars, I can make them sort of break down the choices made in the research process. The authors write this but where does it come from, how have they reached those results. What do you think? (Educator #3)

Additionally, the educators used scientific articles and course books to make the students critical of sources and stimulated them to give reasons for why they chose a certain reference. Reflections with the students could also revolve around why different books have contradictory facts or why some facts are less applicable in clinical situations. When teaching strategies took their point of departure in scientific articles or course books, the educators claimed that guiding questions were needed; however, some gave such questions to the students, whereas others let the students write the questions themselves as a way of cocreating the teaching content.

Another artefact described by the educators, and intended to facilitate critical thinking, was patient cases, which could be either fictive or based on authentic clinical situations that the students or the educators had experienced. In some situations, the educators deliberately presented unclear cases without exact answers to stimulate critical thinking. The cases could also be real cases that constitute deviations from quality care or stories from newspapers about situations in healthcare. To read novels, use pictures, or let the students paint was also presented as starting points for critical dialogues. A different strategy, compared to inviting reflections on situations that had already taken place, was to make the students reflect over a concrete case taking place in the classroom, for example, hygiene routines, or to make the students perform a fictive conversation with a patient.

I consciously include pitfalls in the cases and write them unclearly so that it's possible to interpret them in different ways. That's our goal, that the students should interpret the cases differently. (Educator #7)

Lastly, the educators used written reflections as an artefact to facilitate critical thinking. It could be a written diary based on predefined questions or a chat forum online. The educators also asked the students to write down their reflections both after clinical placements and after teaching strategies, as a way of making them reflect on thoughts and feelings that had arisen.

### 5.5 Using Oneself as a Tool

The educators described how they used themselves as tools in the facilitation of critical thinking through exemplifying their reasoning and thinking and stating that they cannot answer every question. Additionally, they encouraged the students to contradict them during the teaching strategies but have arguments for it, in order to exemplify how to exchange standpoints in a critical dialogue. In cases where more than one educator was present in the classroom, one strategy was to show that they did not always agree but could have different opinions. This was to highlight that there is seldom one exact answer and that critical thinking is necessary to navigate this fact.

The students often think that we hold all the answers, but I am of the opinion that reasoning, such as critical thinking, […] can be beneficial, to show them that different ways of thinking exist. (Educator #3)

The educators also shared their own clinical experiences, shortcomings flaws and mistakes to demonstrate that they are human and to make the students comfortable in sharing their thoughts and reflections. This was a strategy to make the students critical thinkers through self‐reflection, that is, make them assess their own contribution and how they are affected by their experiences, as well as inspiring the students to imagine themselves as registered nurses and how they want to be perceived.

I often try to exemplify from the clinical practice and share a lot about situations that I've been through, and my experience is that this acts as an eye opener for the students because there are often different perspectives and viewpoints. (Educator # 21)

### 5.6 Outcome Space

**FIGURE 2:**
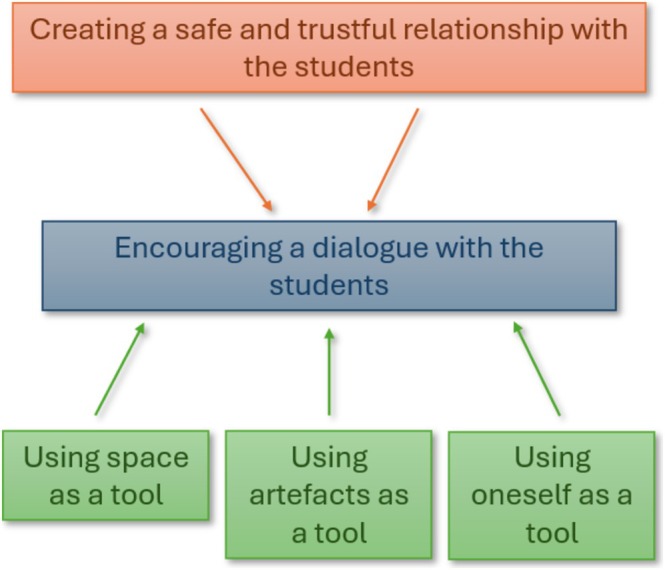
Outcome space.

In phenomenographic studies, the findings are presented as an outcome space that illustrates relations between the identified descriptive studies (Han and Ellis 2019). The outcome space of the current study is presented in Figure 2. The result of the study indicated that the facilitation of critical thinking in nursing students was primarily based on creating a trustful and safe relationship with the students. This relationship was then a precondition for the dialogue between the educator and the students to be encouraged. In the dialogue, the educators used the following tools to aid the conversation: space, artefacts, and themselves.

## 6 Discussion

This phenomenographic study aimed to describe the variation in nurse educators' conceptions of how they facilitate critical thinking in nursing students. What was found to be crucial was creating a safe and trustful relationship with the students, focusing on their strengths and including active encouragement. Based on this, it could be argued that the facilitation of critical thinking in nursing students is not solely dependent upon the implemented teaching strategy. Rather, without the establishment of a relationship, it does not matter what pedagogical theory or activity is chosen, because the facilitation will be difficult to occur. To be more precise, what could be needed is the combination of the two, where the relationship is seen as a possible foundation for the teaching strategy to be built on. This goes in line with relational teachership, which emphasises trustful student–teacher relationships as the basis for learning and students' growth (Ljungblad 2021). Further, human beings are social, and our lives are constituted by relationships; therefore, teaching should be seen as a relational process (ibid.). As described by Hickey and Riddle (2024), when students and teachers meet and interact, they share a location where the education takes place, which is not considered geographical or spatial. Something happens in this intersection between the student and the teacher where the relation is vital for learning to occur (ibid.).

To become critical thinkers, the students could not only be helped by safe relationships but also put their knowledge and their assumptions in a new light and question them. Here, the educators have a vital role in being the guides and facilitators of the dialogue, to strengthen the students' critical thinking processes. This could also be connected to the above reasoning on shared location and space (Hickey and Riddle 2024) because that is where a dialogue between the students and the educator takes place. The importance of a dialogue in the classroom is also highlighted in dialogic education, emphasising student activity and the collective role of creating educational content and thinking together (Cui and Teo 2021, 2023). This educational approach to the facilitation of critical thinking is presented by Cui and Teo (2023), who found that the educators stimulated the students' thinking through counterarguments, making the students think twice by taking the role of the Devil's advocate and, in some situations, not giving an exact answer but turning it back to the group to discuss. Such dialogic strategies are not only in line with the findings of the current study but also illustrate that how educators facilitate critical thinking might not be dependent on the subject taught, since Cui and Teo (2023) pursued their study in a university English reading class. Using dialogic education also changes the role of the educator from one transmitting knowledge to the students to an interactive role collaborating with the students in partnership (Hajhosseiny 2012). In this different role, which is described in the current study, the educator becomes one important tool in using themselves as an example in enacting critical thinking. In a similar way, Raymond et al. (2018) found how educators are role models to the students in applying their critical thinking when communicating with patients and the healthcare team, prioritising and solving clinical problems. But the educators also use themselves as examples in the direct communication with the students through thinking aloud and giving clues to guide the students' thinking process (Raymond et al. 2018). This emphasises that critical thinking is not a subject that can be taught through solely transferring it from the educator to passively being received by the students.

To facilitate critical thinking, a dialogue is helpful, where both educator and students are actively involved in sharing experiences and viewpoints, as well as being open to different opinions. However, to realise such a dialogue, the educators could apply different tools, one of them being to use themselves as an example, but also space and artefacts. This can be connected to Tietjen et al. (2023) and Leijon (2016), who both claim that room, material, and persons interact with each other and are intertwined in educational situations. The classroom is not seen as just a passive place where the teaching takes place, but it can affect relations, participation, and the outcome of teaching. Similarly, educational material and artefacts do not exist parallel to the persons using them; they affect each other, which is known as the sociomaterial perspective (Tietjen et al. 2023). What was emphasised in the current study was that the physical space is not always the preferred one in the facilitation of critical thinking, as some educators perceived the students to be more comfortable in conversations online. One explanation might be today's digital society where the pace of the digitalisation of higher education was speeded up due to the COVID‐19 pandemic starting in 2020. Today, more education is delivered either completely digitally or in hybrid formats, making it possible for students to participate remotely (Reitan et al. 2024). However, the transfer to online education could be criticised in relation to the subject taught, in this case nursing, which is based on personal meetings with patients mostly in a physical setting such as a hospital or nursing home. In the current study, several educators argued that physical meetings with students are necessary to enable the facilitation of critical thinking. In the same way as with patient meetings, in a physical space it is possible to interpret body language, non‐verbal expressions, and reactions, something which was claimed to be necessary for facilitating a dialogue. Connected to the conditions for the educators, Reitan et al. (2024) also found that in the post‐pandemic digital era, more teaching hours are spent on designing and developing digital educational artefacts than on meeting the students face to face in the classroom. As Säljö (2010) argues, technological tools and computers do not always improve education, and educators need to be careful how those tools are implemented. Further, with the growing use of and access to artificial intelligence (AI) in society, in education as well as in healthcare, it becomes more vital that future nurses are well equipped with critical thinking skills to navigate information and to apply it in clinical patient situations. Here, educators play an important role in the facilitation of critical thinking, reflecting on how digital tools could be used in a safe way. For this to be implemented, teaching hours need to be allocated for the educators to meet the students to enable dialogues. In a similar way, as the findings of this study indicate, small group teaching, which aids face‐to‐face interaction with the students, is one component in the facilitation of critical thinking. However, due to shortage of staff, time constraints, and large student groups, the majority of teaching in nursing programmes is conducted as lectures (Hong and Yu 2017). This indicates an organisational problem and obstacle in the facilitation of critical thinking.

## 7 Limitations

One limitation with the current study is that it is a national interview study conducted in Sweden. Consequently, the educators' demographics, context, own pedagogical education, and the responsibilities of nurses can be seen differently from an international perspective. Therefore, to increase the study's transferability, the context and participants' demographics have been outlined as clearly as possible. Including educators from more countries could have caused a possible problem due to comparability in the case of the length of the nursing programme and the academic degree reached by the students. The fact that the interviews departed from the educator's thoughts on Scheffer and Rubenfeld's (2000) definition of critical thinking might have limited their own perceptions of critical thinking and the facilitation in nursing students. Nevertheless, the mind map was considered a mere point of departure for the interviews, with the analysis of data still inductively pursued. A last limitation of the current study is the fact that not all interviews were transcribed verbatim, as six of them were only listened to for verification. This could have made the analysis of the educator's conceptions unequal; however, the authors found no indication of the analysis being less thorough or detailed through listening to the recordings.

## 8 Conclusion and Educational Implications

The strategies and tools found in this study are not a blueprint for how courses in nursing programmes should be designed to facilitate critical thinking in nursing students. They should rather be seen as an approach towards students, which should be infused in all teaching, theoretical as well as practical, and be repeated throughout the education since it takes time for the students to develop critical thinking. In the facilitation of critical thinking, it is crucial for the educators to create an open educational environment incorporating safe and trustful relations with the students. Here, it is possible for the educators to ask reflective questions and invite the students into critical dialogues. However, more research is needed on the students' perspective on how critical thinking should be developed and facilitated in nursing education, as well as on the exploration of the extent to which the described strategies for facilitating critical thinking are bound to a profession or can be used for interprofessional education and practice. Further, the findings of these studies could be used as a foundation for workshops for educators on how critical thinking may be facilitated in nursing education with a focus on relations, dialogues, and learning spaces. As an evaluation, the effect of this intervention could be measured concerning the level of critical thinking in the students before and after.

## Author Contributions

All four authors have substantially contributed to the conception and design of this paper. F.W. collected the data. F.W. and E.C. had the main responsibility for the analysis, and G.B. and A.W. contributed valuable insights and reflections throughout the process. F.W. was responsible for writing the paper, but it was critically revised and supervised by A.W., G.B., and E.C. All four authors gave their approval for the last draft to be published and are accountable for all aspects of the work.

## Conflicts of Interest

The authors declare no conflicts of interest.

## Data Availability

The data that support the findings of this study are available on request from the corresponding author. The data are not publicly available due to privacy or ethical restrictions.
